# One Health Genomic Analysis of Extended-Spectrum β-Lactamase‒Producing *Salmonella enterica*, Canada, 2012‒2016

**DOI:** 10.3201/eid2807.211528

**Published:** 2022-07

**Authors:** Amrita Bharat, Laura Mataseje, E. Jane Parmley, Brent P. Avery, Graham Cox, Carolee A. Carson, Rebecca J. Irwin, Anne E. Deckert, Danielle Daignault, David C. Alexander, Vanessa Allen, Sameh El Bailey, Sadjia Bekal, Greg J. German, David Haldane, Linda Hoang, Linda Chui, Jessica Minion, George Zahariadis, Richard J. Reid-Smith, Michael R. Mulvey

**Affiliations:** Public Health Agency of Canada, Winnipeg, Manitoba, Canada (A. Bharat, L. Mataseje, G. Cox, M.R. Mulvey);; University of Manitoba, Winnipeg (A. Bharat, G. Cox, M.R. Mulvey);; Public Health Agency of Canada, Guelph, Ontario, Canada (E.J. Parmley, B.P. Avery, C.A. Carson, R.J. Irwin, A.E. Deckert, R.J. Reid-Smith);; University of Guelph, Guelph (E.J. Parmley);; Public Health Agency of Canada, St. Hyacinthe, Quebec, Canada (D. Daignault);; Cadham Provincial Laboratory, Winnipeg (D.C. Alexander);; Public Health Ontario Laboratories, Toronto, Ontario, Canada (V. Allen);; Horizon Health Network, Saint John, New Brunswick, Canada (S. El Bailey);; Laboratoire de Santé Publique du Quebec, Sainte-Anne-de-Bellevue, Quebec, Canada (S. Bekal);; Queen Elizabeth Hospital, Charlottetown, Prince Edward Island, Canada (G.J. German);; Queen Elizabeth II Health Sciences Centre, Halifax, Nova Scotia, Canada (D. Haldane);; British Columbia Center for Disease Control, Vancouver, British Columbia, Canada (L. Hoang);; Public Health Laboratory, Edmonton, Alberta, Canada (L. Chiu); University of Alberta, Edmonton (L. Chui);; Roy Romanow Provincial Laboratory, Regina, Saskatchewan, Canada (J. Minion);; Newfoundland and Labrador Public Health and Microbiology Laboratory, St. John’s, Newfoundland, Canada (G. Zahariadis)

**Keywords:** antimicrobial resistance, One Health, genomic analysis, extended-spectrum β-lactamases, ESBL, Salmonella enterica, bacteria, food safety, food-borne infections, food animals, retail meats, pets, zoonoses, Canada

## Abstract

Extended-spectrum β-lactamases (ESBLs) confer resistance to extended-spectrum cephalosporins, a major class of clinical antimicrobial drugs. We used genomic analysis to investigate whether domestic food animals, retail meat, and pets were reservoirs of ESBL-producing *Salmonella* for human infection in Canada. Of 30,303 *Salmonella* isolates tested during 2012–2016, we detected 95 ESBL producers. ESBL serotypes and alleles were mostly different between humans (n = 54) and animals/meat (n = 41). Two exceptions were *bla*_SHV-2_ and *bla*_CTX-M-1_ IncI1 plasmids_,_ which were found in both sources. A subclade of *S. enterica* serovar Heidelberg isolates carrying the same IncI1-*bla*_SHV-2_ plasmid differed by only 1–7 single nucleotide variants. The most common ESBL producer in humans was *Salmonella* Infantis carrying *bla*_CTX-M-65_, which has since emerged in poultry in other countries. There were few instances of similar isolates and plasmids, suggesting that domestic animals and retail meat might have been minor reservoirs of ESBL-producing *Salmonella* for human infection.

Nontyphoidal *Salmonella* is a leading cause of foodborne diarrheal illness. There were an estimated 535,000 human cases of invasive infection with nontyphoidal *Salmonella* and 59,100 deaths in 2017 globally (*1*,*2*). Gastroenteritis is usually self-limiting, but antimicrobial treatment, including ceftriaxone, ciprofloxacin, trimethoprim/sulfamethoxazole, or amoxicillin, might be recommended for severe disease and invasive infections (*3*).

Extended-spectrum cephalosporins are a major class of broad-spectrum antimicrobial drugs and can be hydrolyzed by β-lactamases belonging to molecular class C (AmpC type, such as *bla*_CMY-2_) and molecular class A (ESBLs, such as *bla*_CTX-M_, *bla*_SHV_, and some alleles of *bla*_TEM_) (*4*). ESBLs are a special concern because they sometimes cause reduced susceptibility to fourth-generation cephalosporins, such as cefepime, and they tend to be carried on mobile genetic elements (*5*). ESBLs are susceptible to β-lactamase inhibitors (e.g., clavulanic acid) and cephamycin-type cephalosporins (e.g., cefoxitin). Potential reservoirs of antimicrobial drug resistance are the food chain, the community, hospitals, and the environment (*6*). Ceftiofur was used systematically in the poultry industry in Canada. However, as of 2014, the industry voluntarily eliminated preventive use of antimicrobial drugs that were highly essential, including ceftiofur (*7*). As of December 1, 2018, medically essential antimicrobial drugs are available only by veterinary prescription for use in animals in Canada (*8*).

The most common ESBL-producing *Enterobacterales* are *Escherichia coli* and *Klebsiella pneumoniae* carrying CTX-M enzymes, but ESBL *Salmonella* are observed infrequently (*9*–*11*). Approximately 10% of human clinical isolates of *E. coli* and *K. pneumoniae* were ESBL producing in Canada during 2016 (*9*). Three previous studies of a combined total of >11,000 *Salmonella* isolates from humans and animals/meat in North America identified only 7 ESBL isolates during 2005–2008 (*12*–*14*).

We conducted a genomic study of surveillance isolates collected by the Canadian Integrated Program for Antimicrobial Resistance Surveillance (CIPARS) to evaluate the contribution of food animals, retail meat, and pets toward human infections of ESBL-producing *Salmonella* in Canada during 2012–2016. We also characterized ESBL plasmids by short-read and long-read whole-genome sequencing (WGS).

## Methods

### ESBL Detection

ESBL-producing typhoidal and nontyphoidal *Salmonella* collected from 2012–2016 were identified by CIPARS, which collects human clinical samples from all 10 provincial public health laboratories in Canada. CIPARS also collects animals/meat isolates from farms, abattoirs, and retail stores, and veterinary diagnostic samples from animal health laboratories (*15*). We conducted antimicrobial drug susceptibility testing by using broth microdilution, the Sensititer Automated Microbiology System (Trek Diagnostic Systems Ltd., https://www.trekds.com), and breakpoints established by the Clinical Laboratory Standards Institute (*16*). CIPARS carries out susceptibility testing on all *Salmonella* serotypes from animals/meat sources and 11 serotypes from human samples that are either frequently isolated or frequently multidrug-resistant (4,[5],12,i:-, Dublin, Enteritidis, Heidelberg, Infantis, Kentucky, Newport, Paratyphi A, Paratyphi B, Typhi, and Typhimurium). We performed the combination ESBL disk test (cefotaxime or ceftazidime alone or in combination with clavulanic acid) on human isolates belonging to serotypes that were identified as ESBL producing in animals/meat that were not initially tested by broth microdilution (Anatum, Worthington Agona, Albany, Bredeney, Brandenburg, California, Derby, Ohio, and Ouakam).

We subjected isolates that had a ceftriaxone MIC >0.5 mg/L to the ESBL disk test and a β-lactam PCR to detect *bla*_CMY-2_, *bla*_TEM_, *bla*_CTX-M_, *bla*_SHV_, and *bla*_OXA_ as described (*17*). Isolates were selected for WGS if they showed positive results in the ESBL disk test or if they contained *bla*_CTX-M_, *bla*_SHV_, or *bla*_OXA_. To capture ESBL variants of *bla*_TEM_, we also sequenced isolates that were positive for *bla*_TEM_ by PCR but lacked CMY-2 or another ESBL-hydrolyzing enzyme.

### Short-Read WGS

We subjected potential ESBL-producing *Salmonella* to short-read sequencing. We extracted DNA by using the Epicenter Complete DNA and RNA Extraction Kit (Illumina, https://www.illumina.com). We prepared libraries by using the Nextera XT Kit and sequenced them on the Miseq Platform with the Miseq Reagent v3 600 Cycle Kit (both from Illumina) The average genome coverage was 95× (range 68×‒147×), and the average N50 (length of the shortest contig in the group of longest contigs that together represent >50% of genome assembly) of assemblies was 411,319 bp (range 79,379‒740,528 bp), indicating high quality of sequencing and assemblies.

### Long-Read WGS

We conducted long-read WGS on a subset of isolates by using the MinION Platform (Oxford Nanopore Technologies, https://nanoporetech.com). We prepared libraries by using the Rapid Barcoding SQK-RBK004 Kit and sequenced them by using R9.4 Flowcells (both from Oxford Nanopore Technologies). All *bla*_SHV-2_ isolates were selected for long-read sequencing because this gene was commonly detected in humans (n = 12) and animals/meat (n = 15). If an ESBL variant was observed >5 times in 1 source (humans or animals/meat), we selected a convenience sample of 3 isolates from that source for long-read sequencing (*bla*_CTX-M-55_ and *bla*_CTX-M-65_ in humans and bla_CTX-M-1_ in animals/meat). If the ESBL enzyme was observed <5 times in 1 source, we selected 1 isolate for long-read sequencing (*bla*_SHV-2_, *bla*_CTX-M-1_, *bla*_CTX-M-3_, *bla*_CTX-M-9_, *bla*_CTX-M-14_, and *bla*_CTX-M-15_ in humans and *bla*_CTX-M-55_ in animals/meat).

### Assembly and Alignment

We assembled short reads by using SPAdes Galaxy version 3.11.1 (*18*). We conducted plasmid assembly by using Unicycler version 0.4.7, which combines the accuracy of short reads with the scaffolding of long reads (*19*). Determinants of antimicrobial drug resistance and plasmids were detected by using the Public Health Agency of Canada StarAMR Tool (*20*), which incorporates the ResFinder, PointFinder, and PlasmidFinder databases (*21*,*22*). We created plasmid alignments by using the web-based GView server (https://www.server.gview.ca) parameters: minimum length 150 and minimum nucleotide identity 98%. If an alignment included only plasmids that were closed by long-read sequencing, we used the pangenome feature of GView, which displays all content for all plasmids. If an alignment included any samples that were subjected only to short-read sequencing (fragmented assemblies), we used the BLAST atlas feature of GView, which displays only homology to a closed reference plasmid. We described plasmids as being similar if they displayed >95% nucleotide identity over >90% of the length of the plasmid.

### Phylogenetic Trees

We used the single-nucleotide variant (SNV) phylogenomics (SNVPhyl) pipeline (https://snvphyl.readthedocs.io/en/latest) to build genomic dendrograms based on SNVs in the core genome (*23*). In brief, we mapped reads to a reference genome by using SMALT (https://bio.tools/smalt). We called variants by using mpileup (http://comailab.genomecenter.ucdavis.edu/index.php/mpileup) and Freebayes (https://www.geneious.com/plugins/freebayes), and used consensus SNVs to build the dendrogram by using PhyML (*24*) and the generalized time reversible model. Parameters used were minimum coverage 10, minimum mapping quality 30, and SNV density filtering > 2 SNVs/20 base window. SH-like branch support values >0.95 were considered to be strong support for internal branches (*24*).

### Accession Identifiers

We deposited read data for all isolates in this study to the National Center for Biotechnology Information Short Read Archive under BioProject PRJNA740259. We provided the BioSample identification for the isolates (Appendix Table).

## Results

### WGS of ESBL-Producing *Salmonella*

During 2012–2016, the prevalence of class A ESBL enzymes in Canada was 0.35% in humans (54 ESBL-positive/15,401 screened) and 0.31% in the animals/meat and pet samples (41 ESBL-positive/14,923 screened) within CIPARS. The animals/meat-source *Salmonella* were from turkey/turkeys (refers to meat/animal; 5 from meat, 17 from animals), pigs (n = 11, all animals), cattle (n = 4, all animals), chicken/chickens (1 from meat, 2 from animals), and domestic cat (n = 1) (Table 1). Thus, only 6 samples were from meat, and the remaining 36 samples were from animals, including healthy animals on farms (n = 11, 26.8%), and veterinary clinical samples from sick animals (n = 24, 68.5%). The human-source *Salmonella* were from stool (n = 48), blood (n = 2), urine (n = 2), and unknown sources (n = 2). We provided detailed information on all isolates (Appendix Table).

**Table 1:** Animals/meat hosts carrying ESBL-producing *Salmonella* sp., Canada*

Source	ESBL recovery, no. positive/no. tested (%)	Meat	Farm	Veterinary
Total	41/13,120 (0.31)	6	11	24
Chicken/chickens	3/7,239 (0.04)	1	1	1
Cat (domestic)	1/22 (4.5)	NA	NA	1
Cattle	4/981 (0.4)	0	0	4
Pigs	11/3,312 (0.33)	0	4	7
Turkey/turkeys	22/1,416 (1.55)	5	6	11

*ESBL, extended-spectrum β-lactamase; NA, not applicable.

### ESBL Serotypes and Alleles

ESBLs were detected in a variety of *Salmonella* serotypes from human sources (54 isolates belonging to 11 serotypes) (Table 2) and animals/meat sources (41 isolates belonging to 14 serotypes) (Table 3). In humans, the most common ESBL-producing serotypes were Heidelberg (n = 16; 29.6%), Infantis (n = 15; 27.8%), Typhimurium (n = 7; 13.0%) and 4,[5],12,i:- (n = 5; 9.3%) (Table 2). In the animals/meat sources, the most common ESBL-producing serotypes were Albany (n = 15; 36.6%), Heidelberg (n = 6; 14.6%), and Agona (n = 4; 9.8%) (Table 3). Overall, *Salmonella* Heidelberg was the most commonly detected ESBL-producing serotype (n = 22; 23.2%) in the study.

**Table 2:** Distribution of ESBL-producing *Salmonella* serotypes from human sources, Canada*

Serotype	Serotype subtotal, no. (%)	*bla*_CTX-M-1_	*bla*_CTX-M-3_	*bla*_CTX-M-9_	*bla*_CTX-M-14_	*bla*_CTX-M-15_	*bla*_CTX-M-55_	*bla*_CTX-M-65_	*bla*_SHV-2_	*bla*_SHV-5_
Allele subtotal	54	4 (7.4)	2 (3.7)	3 (5.6)	3 (5.6)	5 (9.3)	**6 (11.1)**	**18 (33.3)**	**12 (22.2)**	1 (1.9)
4,[5],12:i:-	5 (9.3)	0	0	0	1	1	3	0	0	0
Enteritidis	2 (3.7)	0	1	0	0	0	1	0	0	0
Heidelberg	**16 (29.6)**	3	0	0	1	0	0	0	**12**	0
Infantis	**15 (27.7)**	0	1	0	0	0	0	**14**	0	0
Newport	2 (3.7)	0	0	0	0	1	0	0	0	1
Typhimurium	**7 (1)**	1	0	2	0	0	0	4	0	0
Typhimurium O:5-	2 (3.7)	0	0	1	0	0	1	0	0	0
Other serotypes†	5 (9.3)	0	0	0	1	3	1	0	0	0

*Bold indicates >5 occurrences. ESBL, extended-spectrum β-lactamase. †Other serotypes include 1 each of Agona, Chester, Concord, Minnesota, and Poona.

**Table 3:** Distribution of ESBL-producing *Salmonella* serotypes from animals/meat sources, Canada*

Serotype	Serotype subtotal, no. (%)	*bla*_CTX-M-1_	*bla*_CTX-M-55_	*bla*_SHV-2_	*bla*_SHV-12_
Allele subtotal	41	**19 (46.3)**	1 (2.4)	**15 (36.6)**	**6 (14.6)**
4,[5],12:i:-	1 (2.4)	0	1	0	0
4,12:-:-	1 (2.4)	0	0	0	1
Agona	4 (9.8)	0	0	4	0
Albany	**15 (36.6)**	**15**	0	0	0
Brandenburg	2 (4.9)	0	0	1	1
California	2 (4.9)	0	0	2	0
Derby	2 (4.9)	0	0	2	0
Heidelberg	**6 (14.6)**	0	0	2	4
Ohio	2 (4.9)	0	0	2	0
Ouakam	2 (4.9)	2	0	0	0
Other serotypes†	4 (9.8)	2	0	2	0

*Bold indicates >5 occurrences. ESBL, extended-spectrum β-lactamase. .†Other serotypes include 1 each of Anatum, Bredeney, Infantis, and Worthington.

A wider diversity of ESBL enzymes were found in human sources (9 alleles) than in animals/meat sources (4 alleles). In human-source *Salmonella*, the most common ESBLs were *bla*_CTX-M-65_ (n = 18, 33.3%), *bla*_SHV-2_ (n = 12, 22.2%), and *bla*_CTX-M-55_ (n = 6, 11.1%) (Table 2). Human-source *Salmonella* also carried *bla*_CTX-M-1_, *bla*_CTX-M-3_, *bla*_CTX-M-9_, *bla*_CTX-M-14_, and *bla*_CTX-M-15_ and *bla*_SHV-5_ at lower frequencies. Most animals/meat-source isolates contained either *bla*_CTX-M-1_ (n = 19, 46.3%) or *bla*_SHV-2_ (n = 15, 36.6%); the remaining isolates contained either *bla*_SHV-12_ (n = 6) or *bla*_CTX-M-55_ (n = 1) (Table 3). Thus, the *bla*_SHV-2_ gene was detected in 20% of ESBL *Salmonella* from human sources and in 33.3% of ESBL *Salmonella* from animals/meat sources. Except for isolate 12-0820, all *bla*_SHV-2_ isolates carried a known L31Q substitution, which is sometimes referred to as *bla*_SHV-2a_.

### Drug Resistance Profiles

Resistance to ampicillin, streptomycin, sulfisoxazole, and tetracycline (ASSuT) was commonly observed in ESBL-producing *Salmonella* isolates from both sources (Table 4). For human source isolates, 25 (46.2%) demonstrated the ASSuT and chloramphenicol resistance pattern. For animals/meat sources, 12 (29.3%) isolates displayed the ASSuT and gentamicin resistance pattern. Although *bla*_SHV_ and *bla*_CTX-M_ alleles conferred ceftriaxone resistance (MIC resistance breakpoint >4 mg/L), isolates that had *bla*_SHV_ showed MICs of 4–8 mg/L, and isolates that had *bla*_CTX-M_ showed 8-fold higher MICs of 32–64 mg/L. Intermediate or outright resistance to ciprofloxacin was frequently observed in human-source ESBL-producing *Salmonella* (n = 31, 57.4%) but not in animals/meat sources.

**Table 4:** Phenotypic susceptibility and genetic resistance determinants for 13 antimicrobial drugs in *Salmonella* sp. Canada

Antimicrobial drug	Human source, n = 54			Animals/meat source, n = 41	
	No. resistant (%)*	Genetic determinants†		No. resistant (%)*	Genetic determinants†
Amoxicillin/clavulanic acid	0	None		6 (14.6)	*bla*_CMY-2_ (n = 6)
Ampicillin	54 (100)	*bla*_CTX-M-1_, *bla*_CTX-M-3_, *bla*_CTX-M-9_, *bla*_CTX-M-14_, *bla*_CTX-M-15_, *bla*_CTX-M-55_, *bla*_CTX-M-65_, *bla*_SHV-2_, *bla*_SHV-5_ (n = 54)		41 (100)	*bla*_CTX-M-1_, *bla*_CTX-M-55_, *bla*_SHV-2_, *bla*_SHV-12_ (n = 41)
Azithromycin	0	None		1 (2.4)	*mphA* (n = 1)
Chloramphenicol	29 (53.7)	*floR*, *catA*, *catB*, *cmlA* (n = 27)		7 (17.1)	*floR* (n = 5)
Iprofloxacin	31 (57.4)	GyrA D87Y or D87G, *qnrA1*, *qnrB1*, *qnrS1*, *aac(6')-Ib-cr* (n = 31)		3 (7.3)	*qnrB2, qnrS1* (n = 3)
Ceftriaxone	54 (100)	*bla*_CTX-M-1_, *bla*_CTX-M-3_, *bla*_CTX-M-9_, *bla*_CTX-M-14_, *bla*_CTX-M-15,_ *bla*_CTX-M-55_, *bla*_CTX-M-65_, *bla*_SHV-2_, *bla*_SHV-5_ (n = 54)		41 (100)	*bla*_CTX-M-1_, *bla*_CTX-M-55_, *bla*_SHV-2_, *bla*_SHV-12_ (n = 41)
Cefoxitin	1 (1.9)	None		7 (17.1)	*bla*_CMY-2_ (n = 6)
Gentamicin	13 (24)	*aac(**3**)-IIa*, *aac-(**3**)-IId*, *aac(**3**)-IVa*, *aac(**3**)-Vla*, and *rmtB* (n = 25)		24 (58.5)	*aac(**3**)-IId, aac(**3**)-Vla, aac(6')-Iy, and aac(6')-IIc* (n = 23)
Nalidixic acid	20 (37)	GyrA D87Y or D87G, *qnrS1* (n = 20)		0	
Sulfisoxazole	35 (64.8)	*sul1*, *sul2*, *sul3* (n = 35)		26 (63.4)	*sul1*, *sul2*, *sul3* (n = 26)
Streptomycin	28 (51.9)	*aadA1*, *aadA2*, *ant(3”)-Ia*, *ant(3”)-Ib*, *aph(3”)-Ib*, and *strA* (n = 31)		23 (56.1)	*aadA1*, *aadA2*, *ant(3”)-Ia*, *ant(3”)-Ib*, and *strA* (n = 29)
Sulfamethoxazole/trimethoprim	26 (48.1)	*dfrA1*, *dfrA12*, *dfrA14*, *dfrA16*, *dfrA18*, *dfrA23* (n = 26)		12 (29.3)	*dfrA1*, *dfrA14*, *drfA18* (n = 12)
Tetracycline	43 (79.6)	*tetA* and *tetB* (n = 40)		21 (51.2)	*tetA*, *tetB*, and *tetD* (n = 21)

*Where available, resistance was interpreted according to Clinical Laboratory Standards Institute breakpoints; for azithromycin, the *National Antimicrobial Resistance Monitoring System* NARMs breakpoint of 32 mg/L was used; for ciprofloxacin, both intermediate and full resistance were included. †n value in parentheses indicates number of isolates that contained >1 genetic determinant of resistance.

There was general agreement between resistance phenotypes and genotypes (Table 4). CMY-2, but not ESBLs, confer resistance to clavulanic acid; 6 animals/meat isolates had resistance to amoxicillin/clavulanic acid and all contained *bla*_CMY-2_. One human-derived isolate of *Salmonella* 4,[5],12:i:- had the mobile colistin resistance gene *mcr-3.2*, along with *bla*_CTX-M-55_ and other resistance genes conferring resistance to 8 classes of antimicrobial drugs; the isolate had been described (*25*). Agreement between phenotype and genotype for gentamicin was lower; 12/25 isolates that had predicted gentamicin resistance contained the *aac (**3**)-IVa* gene conferring MICs of 4–8 mg/L, which is 1 or 2 dilutions below the resistance breakpoint of MIC >16 mg/L and accounted for most of the discrepancy. In general, disagreements between susceptibility phenotype and genotype might be caused by resistance genes or mutations that are currently unknown.

### Phylogenomic Relatedness of Strains

**Figure 1:**
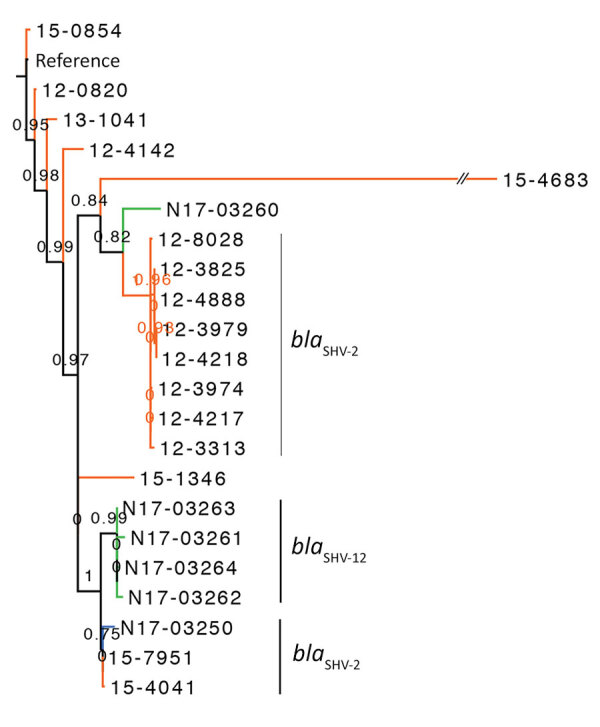
Phylogenetic dendrogram of *Salmonella enterica* serovar Heidelberg containing extended-spectrum β-lactamase genes, Canada. The maximum-likelihood dendrogram was created by using the single-nucleotide variant (SNV) phylogenomics (SNVPhyl) pipeline (https://snvphyl.readthedocs.io/en/latest) based on SNVs in the core genome. The reference genome is *Salmonella* Heidelberg strain 12-4374 (GenBank accession no. CP012924.1). The tree is based on a core genome that represents 94% of the reference genome. Numbers along branches indicate branch support values. *Salmonella* Heidelberg containing extended-spectrum β-lactamases were from animals (green, n = 5), food (blue, n = 1), and humans (orange, n = 16). Extended-spectrum β-lactamase genes are indicated to the right of the 3 largest clusters. The dataset comprises 394 SNVs, and SH-like branch support values are displayed.

We created maximum-likelihood phylogenetic trees based on SNVs in the core genome for each serotype. The *Salmonella* Heidelberg phylogenetic tree showed that ESBL-producing isolates from human and animals/meat sources were genetically distinct, with some exceptions (Figure 1). Closely related *Salmonella* Heidelberg (defined as <20 SNVs) carrying *bla*_SHV-2_ were identified from chicken thighs (N17-03250 isolated in western Canada during 2013) and humans (15-7951 and 15-4041 isolated in western Canada during 2015); these isolates differed by only 1–7 SNVs. The branch support for these 3 isolates was not strong (SH-like value 0.76). However, the isolates carried similar type A IncI1-*bla*_SHV-2_ plasmids (described in the ESBL Plasmids Section). The 3 isolates carrying *bla*_SHV-2_ were also genetically similar to 4 isolates carrying *bla*_SHV-12_, differing by only 9–14 SNVs.

**Figure 2:**
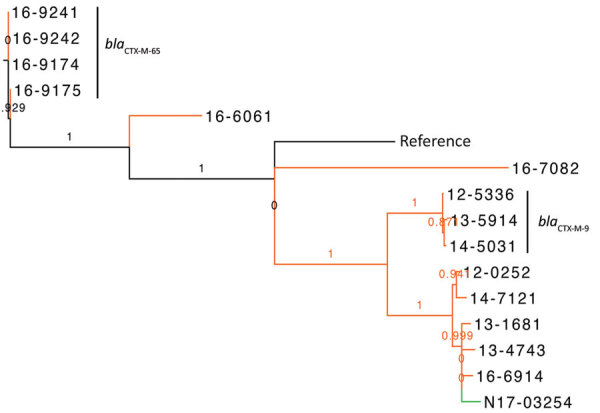
Phylogenetic dendrogram of extended-spectrum β-lactamase‒producing *Salmonella enterica* serovars Typhimurium and 4,5,12,i:-, Canada. The maximum likelihood dendrogram was created by using the single-nucleotide variant (SNV) phylogenomics (SNVPhyl) pipeline (https://snvphyl.readthedocs.io/en/latest) based on SNVs in the core genome. The reference genome is *Salmonella* Typhimurium strain LT2 (GenBank accession no. NC_003197.2). The tree is based on a core genome that represents 96% of the reference genome. Numbers along branches indicate branch support values. Sample N17-03254 was a clinical isolate from a sick pig (green), and all other samples were from human sources (orange, n = 14). Extended-spectrum β-lactamase genes are indicated to the right of the 3 largest clusters. The dataset comprises 1,599 SNVs, and SH-like branch support values are displayed.

The phylogenetic tree for *Salmonella* Typhimurium and closely related serovars Salmonella 4,5,12,i:- and *Salmonella* Typhimurium var. O:5 showed that isolates from human and animals/meat sources were genetically distinct with 1 potential exception (Figure 2). Among *Salmonella* 4,[5],12,i:- carrying *bla*_CTX-M-55_, 1 clinical isolate from a sick pig (N17-03254 isolated in central Canada during 2015) differed by 55–80 SNVs from 3 human isolates (13-1681, 13-4743, and 16-6914, isolated in central and western Canada during 2013 and 2016) (Figure 2). More epidemiologic studies are needed to interpret whether 55–80 SNVs indicate genetic relatedness. However, the clustering of isolates from human and pig was strongly supported (SH-like value 0.99).

**Figure 3:**
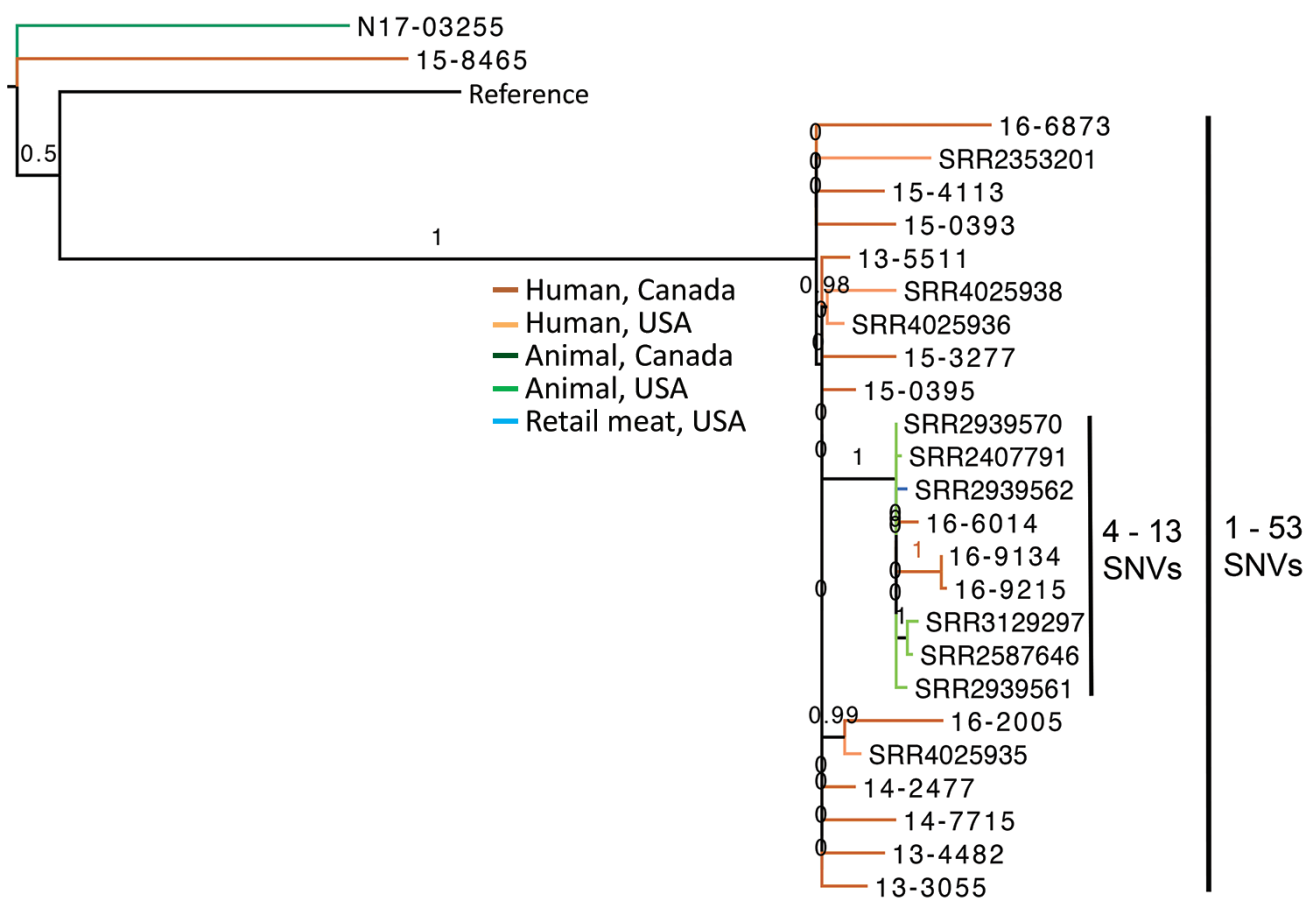
Phylogenetic dendrogram of extended-spectrum β-lactamase‒producing *Salmonella* *enterica* serovars Infantis from Canada and the United States. Isolates from the United States are from Tate et al. (*26*). The maximum-likelihood dendrogram was created by using the single-nucleotide variant (SNV) phylogenomics (SNVPhyl) pipeline (https://snvphyl.readthedocs.io/en/latest) based on SNVs in the core genome. The reference genome was *Salmonella* Infantis strain 15-SA01028 (GenBank accession no. CP026660.1). The tree is based on a core genome that represents 97% of the reference genome. Numbers along branches indicate branch support values. *Salmonella* Infantis containing extended-spectrum β-lactamases were isolated from human sources in Canada (dark orange), human sources from the United States (light orange), a cat from Canada (dark green), poultry or dairy at slaughter from the United States (light green) or retail meat from the United States. Isolate N17-03255 from a cat contained SHV-2, isolate 15-8465 from a human contained CTX-M-3, and all other isolates contained CTX-M-65. The dataset comprises 491 SNVs, and SH-like branch support values are displayed.

*Salmonella* Agona and *Salmonella* Infantis were the only other serotypes in which ESBL-producing isolates were detected in both sources. Phylogenetic trees of *Salmonella* Agona did not show evidence of transmission between animals/meat and humans because isolates from the 2 sources differed by >77 SNVs. The United States and other countries have noted the emergence of *Salmonella* Infantis carrying CTX-M-65 on the plasmid of emerging *Salmonella* Infantis in humans and food animals, especially in poultry (*26*). For comparison, we included genome sequences of *Salmonella* Infantis from human, food animals, and retail chicken from the study by Tate et al. in our phylogenetic tree (*26*). The major clade of the tree comprised CTX-M-65‒containing isolates from both studies whereby isolates differed by 1‒53 SNVs. Three isolates from human sources in Canada collected during 2016 were closely related to isolates from retail chicken, chicken at slaughter, and dairy cow at slaughter collected in the United States during 2014–2015, differing by only 4–13 SNVs and clustering on a strongly supported branch (SH-like value 1.0) (Figure 3). One isolate each from Canada from a cat (N17-03255) carrying SHV-2 and a human (15-8465) carrying CTX-M-3 did not cluster with the CTX-M-65-containing isolates.

### ESBL Plasmids

Although the ESBL serotypes were mostly different between humans and animals/meat isolates, it is possible that ESBL plasmids were similar because plasmids can be transmissible between serotypes. We produced complete plasmid sequences for a subset of isolates.

**Figure 4:**
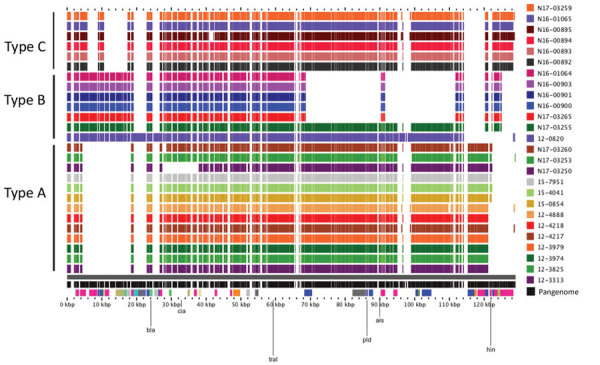
Alignment of *Salmonella* bla_SHV-2_ plasmids from human and animals/meat sources, Canada. Closed plasmids were produced by hybrid assembly of short and long read sequencing by using Unicycler (https://bio.tools/unicycler). Plasmids were aligned by using the pangenome feature of the GView server (https://server.gview.ca). Animals/meat sample identifications start with the letter N, and human sample identifications start with a 2-digit number. Plasmids were classified as Type A, B, or C based on their resistance gene profiles and overall similarity. All plasmids belong to the IncI1 incompatibility group.

The *bla*_SHV-2_ genes from human sources were all from *Salmonella* Heidelberg, and the animals/meat isolates were from a variety of serotypes, including Agona, Anatum, Brandenburg, California, Derby, Heidelberg, Infantis, and Ohio (Table 2). All *bla*_SHV-2_ genes were carried on IncI plasmids, which we categorized into 3 types (types A, B, and C) on the basis of their resistance gene profiles and overall genetic content (Figure 4). Similar *bla*_SHV-2_ plasmids were found in isolates from humans and animals/meat (>95% nucleotide identity over >90% plasmid length).

The type A plasmids carried *bla*_SHV-2_ and *tetA* resistance genes and were found in humans (n = 10), chicken(s) (n = 2, one each from meat and animal), and pig (n = 1 from animal) (Figure 4). Three of the type A plasmids were from *Salmonella* Heidelberg isolates that differed by only 1–7 SNVs (N17-03250 isolated from chicken thighs during 2013; and 15-7951 and 15-4041 isolated from humans during 2015). Thus, these 3 isolates were genetically closely related and contained similar plasmids (99.9% nucleotide identity over 91% of the length of the plasmid). The type B plasmids carried *aac (**3**)-VIa*, *ant(3′′)-Ia, bla*_SHV-2_, and *sul1* and were found in a human (n = 1), a domestic cat (n = 1), and turkey/turkeys (n = 5). The type B plasmid from a human (12-0820 in *Salmonella* Heidelberg) was most similar to the plasmid from the domestic cat (N17-03255 in *Salmonella* Infantis) with 99.9% nucleotide identity over 93% of the length of the plasmid. Finally, the type C plasmids carried *aadA1*, *bla*_SHV-2_, *dfrA1*, and *sul1*. The type C plasmids were isolated from pigs on farms and from sick pigs.

**Figure 5:**
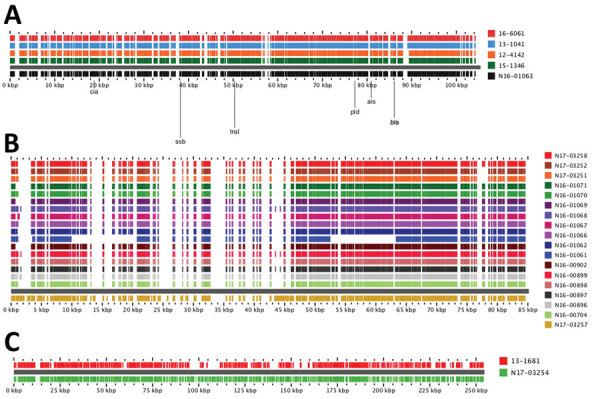
Alignment of *Salmonella bla*_CTX-M_ plasmids from human and animals/meat sources, Canada. Alignments of *bla*_CTX-M-1_ IncI1 (A), *bla*_CTX-M-1_ IncN (B), and *bla*_CTX-M-55_ IncN (C) plasmids are shown. Plasmids were aligned by using the BLAST feature of the GView server (https://server.gview.ca) and representative closed plasmids (bottom-most plasmid in each alignment) from this study. Animals/meat sample identifications start with the letter N, and human sample identifications start with a 2-digit number.

The *bla*_CTX-M-1_‒containing plasmid was the most common ESBL in animals/meat isolates and was occasionally observed in human isolates. One *bla*_CTX-M-1_ plasmid from serotype *Salmonella* Worthington identified from a pig (N16-01063 isolated in western Canada during 2013) and all *bla*_CTX-M-1_ plasmids from *Salmonella* Heidelberg (n = 3) and *Salmonella* Typhimurium (n = 1) from human sources in various years were carried on similar IncI plasmids (Figure 5, panel A). Of 119 coding sequences on the *bla*_CTX-M-1_ IncI plasmid from pig (N16-01063), all but 1 coding DNA sequence was present on the human plasmids. The plasmid had only 75% nucleotide identity to the previously reported R64 reference plasmid (*27*). However, in the National Center for Biotechnology Information database (https://www.ncbi.nlm.nih.gov), there were matches to *E. coli* plasmids (e.g., accession no. CP007651.1).

In the remainder of animals/meat isolates harboring *bla*_CTX-M-1_, the gene was carried on an IncN plasmid (Figure 5, panel B). IncN *bla*_CTX-M-1_ plasmids were similar between isolates except for N16-01061, which was missing ≈20 kb. A representative *bla*_CTX-M-1_ IncN plasmid from isolate N17-03257 had >99.5% nucleotide identity to plasmids from *E. coli* O16:H48 (accession no. CPO34186.1) and *Salmonella* Bredeney (accession no. CPO43184.1). The *bla*_CTX-M-55_ was found on IncN plasmids in *Salmonella* 4,[5],12:i:- in 1 isolate each from human and animals/meat source. However, of 291 coding DNA sequences on the plasmid from a pig isolate (N17-03254), only 242 (83%) were present on the plasmid from the human isolate (13-1681) (Figure 5, panel C).

The most common combination of ESBL *Salmonella* serotype and allele in humans in this study was *Salmonella* Infantis carrying *bla*_CTX-M-65_ (n = 14). The *bla*_CTX-M-65_ IncFIB plasmid was almost identical to a plasmid that the *National Antimicrobial Resistance Monitoring System (*NARMS) has reported to be emerging in the United States in humans and chickens (*26*,*28*). A representative closed plasmid from isolate 15-4113 in this study had 99.1% nucleotide identity with the reported NARMS plasmid (accession no. NZ_CP016407). Of 14 isolates of *Salmonella* Infantis carrying CTX-M-65 detected in Canada, 9 isolates contained 11 antimicrobial resistance determinants: *aac (**3**)-IVa*, *ant(3′′)-Ia*, *aph(3′′)-Ia*, *aph (**4**)-Ia*, *bla_CTX-M-65_*, *dfrA14*, *floR*, *fosA3*, *gyrA* (D87Y), *sul1*, and *tet(A)* conferring reduced susceptibility or resistance to gentamicin, streptomycin, ampicillin, ceftriaxone, trimethoprim, chloramphenicol, ciprofloxacin, nalidixic acid, sulfisoxazole, and tetracycline. Gene *fosA3* probably confers reduced susceptibility to fosfomycin, but this antimicrobial drug was not tested. All resistance determinants except for *gyrA* (D87Y) were carried on the representative closed plasmid. Two isolates lacked *fosA3*, 2 lacked *fosA3* and *aph (**4**)-Ia*, and 1 lacked *fosA3*, *ant(3′′)-Ia* and *aph(3′)-Ia*.

## Discussion

Health Canada has classified third-generation and fourth-generation cephalosporins as Category I (high importance) antimicrobial drugs based on their role in human medicine. However, Category I antimicrobial drugs are still used in food animals with a veterinary prescription with some restrictions (*8*,*29*). The frequency of ESBL-producing Enterobacterales continues to increase in humans, especially in *E. coli* and *K. pneumoniae* (*30*). In this study, the frequency of recovery of ESBL-producing *Salmonella* (i.e., no. ESBL-producing isolates/total no. isolates) during 2012‒2016 was low (0.35% from humans and 0.31% from animals/meat). Recent studies in China have reported a much higher frequency of recovery of ESBL-producing *Salmonella* from food (9.7%) and food animals (17.7%) (*31*,*32*).

In our study, 76% of ESBL-producing *Salmonella* causing human infections and 49% from animals/meat isolates harbored CTX-M, and the remainder harbored SHV. During the 1990s, global outbreaks of ESBL-producing *Enterobacterales* were mainly caused by *K. pneumoniae* carrying SHV and TEM enzymes; since then, CTX-M enzymes have increased rapidly and are now the most common ESBL enzymes (*33*).

For ESBL-related infections in humans, CTX-M-14 and CTX-M-15 are the most common ESBLs in *E. coli* (*10*,*33*). However, in our study, these 2 alleles were infrequently observed in *Salmonella*. In our study, *Salmonella* Infantis carrying *bla*_CTX-M-65_ was the most common ESBL-producer detected from human infections in Canada. In the United States, although ESBL-producing *Salmonella* are rare, *Salmonella* Infantis carrying *bla*_CTX-M-65_ is emerging in human infections and in poultry (*26*,*28*). *Salmonella* Infantis containing *bla*_CTX-M-65_ is also emerging in humans and poultry in other countries, including Italy, England and Wales, Israel, Peru, and Ecuador (*34*–*38*).

The *bla*_CTX-M-65_ is carried on a large IncFIB plasmid termed plasmid of emerging *Salmonella* Infantis along with other resistance determinants. The *bla*_CTX-M-65_ plasmid detected in Canada was almost identical to the IncFIB *bla*_CTX-M-65_ plasmid that was reported in the United States (*25*,*27*). This plasmid is especially concerning because it is transferrable and it carries <10 genes encoding resistance to ampicillin, ceftriaxone, chloramphenicol, gentamicin, nalidixic acid, streptomycin, sulfisoxazole, tetracycline, and trimethoprim. *Salmonella* Infantis containing *bla*_CTX-M-65_ was found exclusively from human infections in our study, but human isolates from Canada collected during 2016 were closely related to isolates from humans, chicken, and dairy cow from the United States that were collected during 2014. The human cases in Canada might have been imported through retail sources, or travel, or might have been acquired from domestic food commodities that were not sampled by CIPARS during the study. Continued surveillance is needed to detect potential emergence of ESBLs in food animals, meat, and other food commodities in Canada.

In animals/meat isolates, *bla*_CTX-M-1_ was the most common allele detected and is the most common allele in animals/meat sources from western Europe (*33*,*39*). The allele *bla*_CTX-M-27_ is emerging in China and Vietnam and was detected in the United States, but this allele was not observed in Canada during this study (*40**,**41*).

The *Salmonella* ESBL alleles and serotypes were mostly different between humans and domestic animals/meat sources during the study period. A meta-analysis of risk factors for fecal ESBL colonization identified recent antimicrobial drug use and international travel as the 2 major risk factors (*42*). CIPARS does not collect information on human travel or imported foods, but these factors might contribute to ESBLs in humans in Canada. Although ESBLs were not detected in typhoidal *Salmonella* during the study period, several cases of extensively drug resistant *Salmnella* Typhi containing *bla*_CTX-M-15_ were imported into Canada during 2018 and 2019 by patients who had traveled to Pakistan, where a large outbreak is ongoing (*43*). Pets might be another reservoir of ESBL-producing bacteria. Although CIPARS examined only 22 pet samples in this study, we detected a *bla*_SHV-2_ plasmid in a cat that was almost identical to a plasmid from a human isolate.

In summary, ESBL-producing *E. coli* and *K. pneumoniae* are a healthcare challenge because treatment options are limited (*30*). Although the frequency of recovery of ESBL-producing *Salmonella* was low in this study, it is essential to continue surveillance because extended-spectrum cephalosporins are a major treatment option for serious or invasive *Salmonella* infections.

Appendix Additional information on One Health genomic analysis of extended spectrum β-lactamase‒Producing *Salmonella enterica*, Canada, 2012‒2016.
